# Effects of geriatric clinical skills training on the attitudes of medical students

**DOI:** 10.1186/1472-6920-14-233

**Published:** 2014-10-23

**Authors:** Adrian O Goeldlin, Andrea Siegenthaler, André Moser, Yvette D Stoeckli, Andreas E Stuck, Andreas W Schoenenberger

**Affiliations:** Division of Geriatrics, Department of General Internal Medicine, Inselspital, Bern University Hospital, and University of Bern, CH-3010 Bern, Switzerland; Institute of Social and Preventive Medicine, University of Bern, Bern, Switzerland

**Keywords:** Aged, Education, Students, Medical, Attitude

## Abstract

**Background:**

Physicians’ attitudes, knowledge and skills are powerful determinants of quality of care for older patients. Previous studies found that using educational interventions to improve attitude is a difficult task. No previous study sought to determine if a skills-oriented educational intervention improved student attitudes towards elderly patients.

**Methods:**

This study evaluated the effect of a geriatric clinical skills training (CST) on attitudes of University of Bern medical students in their first year of clinical training. The geriatric CST consisted of four 2.5-hour teaching sessions that covered central domains of geriatric assessment (e.g., cognition, mobility), and a textbook used by students to self-prepare. Students’ attitudes were the primary outcome, and were assessed with the 14-item University of California at Los Angeles Geriatrics Attitudes Scale (UCLA-GAS) in a quasi-randomized fashion, either before or after geriatric CST.

**Results:**

A total of 154 medical students participated. Students evaluated before the CST had a median UCLA-GAS overall scale of 49 (interquartile range 44–53). After the CST, the scores increased slightly, to 51 (interquartile range 47–54; median difference 2, 95% confidence interval 0–4, P = 0.062). Of the four validated UCLA-GAS subscales, only the resource distribution subscale was significantly higher in students evaluated after the geriatric CST (median difference 1, 95% confidence interval 0–2, P = 0.005).

**Conclusions:**

Teaching that targets specific skills may improve the attitudes of medical students towards elderly patients, though the improvement was slight. The addition of attitude-building elements may improve the effectiveness of future skills-oriented educational interventions.

## Background

When physicians have negative attitudes towards elderly persons, they may provide lower quality care to their elderly patients [1, 2]. Educational interventions designed to change attitudes and increase knowledge about older adults have been tested in many studies, and their effects measured [3–18]. These studies provided evidence that such interventions may improve medical students’ attitudes, but positive effects were not consistently found, and effect sizes were small. Educational interventions can be designed to impart skills, as well as to improve attitudes and increase knowledge, and is possible that skills-oriented educational interventions may be more effective at improving student attitudes toward the elderly.

A recent study systematically reviewed educational interventions designed to improve attitude, knowledge, and skills of medical students in geriatric medicine [19]. The authors concluded that skills-oriented interventions were poorly investigated and that more research is necessary to confirm that skills-specific teaching strategies in geriatrics are effective. Some previous studies used skills as an outcome measure, but not as the main target for intervention [10, 11, 20]. To the best of the authors’ knowledge, no previous study assessed the effect of a geriatric skills-oriented educational intervention with real patients on attitudes of medical students. Our goal was to determine the effect of a geriatric clinical skills training (CST) on the attitudes of medical students in the first year of their clinical training by comparing their before and after scores on the University of California at Los Angeles Geriatrics Attitudes Scale (UCLA-GAS).

## Methods

### Setting and participants

We conducted our research at the University of Bern (UB), one of six medical schools in Switzerland that provides clinical teaching. The medical degree program there lasts 6 years (2 years pre-clinical, and 4 years clinical training). Each UB class (per year) numbers between 150 and 200 medical students and CST in different specialties is an integral component of first year clinical training. All first-year (academic year 2011-2012) UB medical students were eligible to participate in this study. The study was performed in accordance with the Declaration of Helsinki and was approved by the local ethics committee (Kantonale Ethikkommission Bern, Bern, Switzerland).

### Intervention

The intervention was a geriatric CST, which consisted of four 2.5-hour teaching sessions. Students also self-prepared with a textbook distributed prior to the first teaching session. Each teaching session began with a 45-minute interactive introduction to the session topic. Then medical students were broken into small groups of four to five students and the CST continued. The students worked with real patients, at the patients’ bedside, and were supervised by a clinician experienced in geriatric medicine. The four teaching sessions covered these topics: activities of daily living and cognition (first session); mobility and social situation (second session); nutrition, medication, urinary and fecal continence, screening of hearing and vision (third session); and, a complete geriatric assessment, designed to repeat and consolidate (fourth session). The CST used only validated instruments (e.g., Mini Mental State Examination [MMSE] or Basic Activities of Daily Living [BADL]) [21–24]. The textbook contained detailed instructions on how to perform and how to interpret these instruments. During the intervention, in addition to geriatric CST the students had problem-based courses with a focus on pathophysiology in the fields of neurology, endocrinology and psychiatry. Students did not do ward work during the intervention period.

### Assessment of attitude and knowledge

Attitude and knowledge were assessed via questionnaire. To assess attitude, we included a German translation of the UCLA-GAS in the questionnaire [25]. We validated the German translation by back-translating it into English. We slightly adapted one item (“The federal government should reallocate money from Medicare to research on AIDS or pediatric diseases”) so that it was more appropriate to Switzerland. To assess knowledge, we included in the questionnaire four questions formulated by a didactic expert group. Each question had to meet the following two prerequisites: 1) it must be related to a domain covered in the CST training; and, 2) the answer must be found in the textbook provided to medical students before the first teaching session. The experts selected the following three multiple-choice questions: 1) rate a given numerical sequence according to instructions for the calculation task of the MMSE [21]; 2) rate a pentagon drawing according to the instructions for the corresponding task of the MMSE [21]; 3) select among five suggested activities, the activity that did not belong to the BADL [22]. The experts also selected a fourth question, which asked medical students to estimate the time required to perform an MMSE and a clock drawing test in clinical routine. The questionnaire also assessed medical students’ age and sex.

Depending on the CST group the students belonged to, we administered the questionnaire either at the beginning of the first session, or at the beginning of the last session. Thus, each individual medical student answered the questionnaire only once, either at the beginning of the first session or at the beginning of the last session. Because medical students were allocated to the CST groups in alphabetical order based on family names, they completed the questionnaires before or after geriatric CST in a quasi-randomized manner. This methodological approach was selected to avoid bias caused by the same individual repeatedly completing the UCLA-GAS. We informed the students that completing the questionnaire amounted to consent to participate in the study, including an anonymized analysis of their answers.

### Skills assessment

Geriatric skills were assessed by an objective structured clinical examination (OSCE) at the end of the academic year. The OSCE employed standardized patient actors as well as standardized evaluation forms, and was mandatory for all medical students who wished to proceed to the next year of medical education. The OSCE comprised a circuit of 12 stations. Each tested different medical specialty areas, one of which was geriatrics. This study only reports the results from the geriatrics station. An independent academic institution at the University of Bern analyzed OSCE results. The borderline regression method was used to set the pass mark [26].

### Outcomes

The primary outcome of this study was the UCLA-GAS overall scale and its four validated subscales (compassion subscale, resource distribution subscale, medical care subscale, and social value subscale). We compared students evaluated after geriatric CST to students evaluated before geriatric CST [25]. We evaluated knowledge and skills before and after geriatric CST as our secondary outcomes. We hypothesized that attitude, knowledge and skills improve after the intervention. Regarding time estimation for performing the MMSE and the clock drawing test in particular, we hypothesized that the estimated time needed to perform the MMSE and clock drawing test would be lower after the teaching sessions.

### Data analysis

We descriptively analyzed the results of the questionnaire and the OSCE. Descriptive analyses included counts and percentages for categorical variables and median values with their interquartile range (IQR) for continuous variables. For the UCLA-GAS, we calculated the summary scores for the overall scale and its four subscales [8, 25]. Based on the summary score for the overall scale, medical students were dichotomized into two groups: students with positive attitude (score ≥42) and students with negative attitude (score <42) [7]. Answers to the three knowledge-oriented multiple-choice questions were rated as correct or incorrect (i.e., rating the numerical sequence and the pentagon drawing of the MMSE and determining the activity that did not belong to BADL). The knowledge score was calculated as the sum of correct answers to the three knowledge-oriented multiple-choice questions (range 0 to 3). Based on the knowledge score, medical students were dichotomized into two groups: students with good knowledge (score ≥2) and students with little knowledge (score <2). Characteristics between groups were compared by Pearson’s chi-squared test for binary variables and by the Wilcoxon rank sum test for scores. Tests were two-sided. Differences and confidence intervals (CI) in scores from the Wilcoxon test were derived from Hodges-Lehmann estimates [27]. (The Hodges-Lehmann estimate is defined as the median of all possible score differences between two independent groups.) Differences were calculated from scores from students evaluated after the CST minus those evaluated before the CST (positive differences correspond to higher scores in the group evaluated after geriatric CST). Our data were complete, except for five single items of the UCLA-GAS. According to the low missing frequency, missing variables were imputed by its highest frequency value [28]. Data were analyzed with Stata 12.1 (StataCorp LP, College Station, TX, USA).

## Results

### Participants

**Figure 1:**
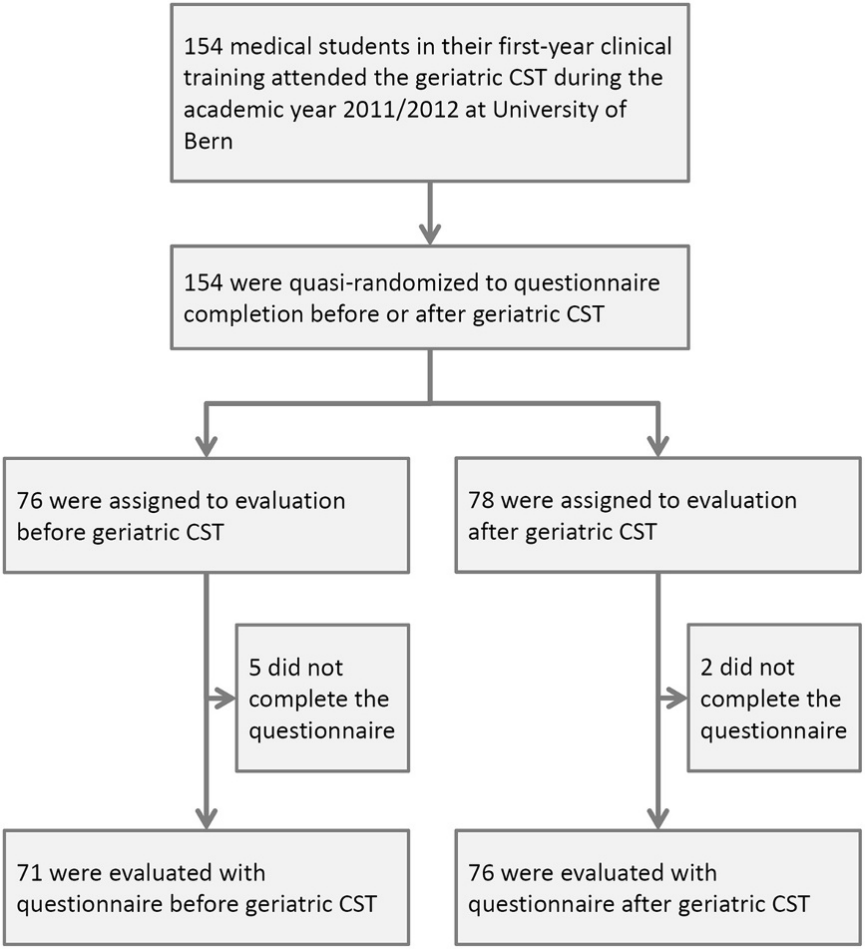
**Flow chart.** CST, clinical skills training.

A flow chart is shown in Figure 1. A total of 154 medical students attended the first-year clinical training in academic year 2011-2012. Of these, seven students (4.8%) did not complete the questionnaire; 147 students (95.2%) participated in the study. Of these, 71 (48.3%) were evaluated before and 76 (51.7%) after the geriatric CST.

The mean age of evaluated students was 22.9 ± 2.1 years (maximum range 20 to 36 years). There were 90 women (61.6%). There were no significant differences in age or sex between students evaluated before and after the geriatric CST.

### Attitude

The median difference of the UCLA-GAS overall scale was 2 points (95% CI 0 – 4, P = 0.062), indicating a higher score in students evaluated after the geriatric CST (Table 1). The nearly significant increase in the overall scale resulted from significant improvement in the resource distribution subscale (Table 1).

**Table 1:** **Attitude and knowledge of medical students before and after the geriatric Clinical Skills Training (CST)**

	Students evaluated before geriatric CST ^a^(N = 71)	Students evaluated after geriatric CST ^a^(N = 76)	Difference (95% CI) ^b^	P-Value ^c^
***Attitude***				
UCLA-GAS				
- Overall scale	49 (44 – 53)	51 (47 – 54)	2 (0 – 4)	0.062
- Compassion subscale	14 (13 – 15)	14 (12 – 16)	0 (-1 – 1)	0.963
- Resource distribution subscale	16 (14 – 17)	17 (15 – 18)	1 (0 – 2)	0.005
- Medical care subscale	11 (10 – 14)	12 (10 – 13)	0 (0 – 1)	0.390
- Social value subscale	7 (6 – 9)	8 (7 – 9)	0 (0 – 1)	0.405
Students with negative attitude	12 (16.9%)	6 (7.9%)	NA	0.096
***Knowledge***				
Knowledge score	1 (-1 – 1)	1 (1 – 3)	2 (0 – 2)	<0.001
Students with little knowledge	32 (45.1%)	15 (19.7%)	NA	0.001
Estimated time needed to perform a MMSE and a clock drawing test in minutes	30 (20 – 30)	20 (15 – 30)	-5 (-10 – 0)	<0.001

*Abbreviations*: CI confidence interval, CST clinical skills training, MMSE Mini Mental State Examination, UCLA-GAS University of California at Los Angeles Geriatrics Attitudes Scale.

^a^Values are medians with interquartile ranges or numbers with percentages.

^b^Difference with 95% confidence interval (CI) based on Hodges-Lehmann estimate [27].

^c^P-Value from two-sided Wilcoxon ranksum test.

NA = not available.

### Knowledge

The median difference in the knowledge score was 2 points (95% CI 0 – 2, P <0.001), which indicated a significantly higher score after the geriatric CST (Table 1). After the geriatric CST, more students correctly rated the pentagon drawing of the MMSE (28.2% before, and 65.8% after the geriatric CST, P <0.001) and correctly identified the activity that was not part of BADL (56.3% before, and 86.8% after the geriatric CST, P <0.001). The proportion of students who correctly rated the numerical sequence of the MMSE calculation task was high before the geriatric CST (76.1%), and was not significantly different from the proportion of students who correctly rated it after the geriatric CST (67.1%). Students evaluated after the geriatric CST estimated they needed significantly less for an MMSE and clock drawing test than those evaluated before the geriatric CST (difference in time estimation -5 minutes, 95% CI -10 – 0 minutes, P <0.001) (Table 1).

### Skills

One student was unable to participate in the OSCE. A total of 153 medical students (99.4%) took the OSCE. Of all participating students, 131 students (85.6%) successfully passed the geriatrics station of the OSCE.

## Discussion

This study shows that after a brief skills-oriented educational intervention medical students’ attitudes towards elderly patients improved and we discuss the possible reasons for this change. The training, which used real patients, and was conducted at the patients’ bedside, seems to have changed negative and reinforced positive attitudes, though the observed effect size on students’ attitudes is small. It appears to be easier to improve knowledge or skills than attitudes.

The increase we observed in the UCLA-GAS overall scale is small—only two points—and there is some question about its relevance. Several previous studies used the UCLA-GAS to evaluate attitude change caused by a teaching intervention for medical students and reported improvement [5, 8, 15]. In these studies, the improvement in the overall scale was also small, ranging 1.12 and 2.52 points. Given these studies’ findings and conclusions, we believe that the change we observed is probably relevant. We support this claim with the following observations. First, some participating medical students had favorable attitudes before they took part in the geriatric CST [8, 25]. The ceiling effect limits the effectiveness of any intervention to improve their scores. Therefore, in a mixed population of students who have both positive and negative attitudes, even a small change in the UCLA-GAS is important, since it indicates a decrease in the proportion of students who have negative attitudes. We observed a similar effect in our study. Second, improvement in the overall scale was attributable to a significant improvement in the resource distribution subscale. Therefore, when statistically significant improvements are apparent only in sections of the UCLA-GAS subscales, even a statistically non-significant improvement to the UCLA-GAS overall scale may be relevant. Of note is that scores on some subscales improved and others did not. Future studies could explore whether some subscales are more amenable to change than others.

The geriatric CST had a clear effect on knowledge and skills. The knowledge score significantly improved after the teaching intervention, and the number of students with little knowledge decreased. Furthermore, the vast majority of students passed the OSCE. Since none of the students had prior experience in geriatric clinical skills, the OSCE result documents that the CST successfully imparted clinical skills. This secondary outcome adds to the current literature [19, 29–33].

Ours was the first study to assess the effect of this kind of educational intervention on the attitudes of medical students, and is valuable because it provides new evidence about the effect of skills-oriented educational interventions on medical student attitudes, knowledge, and skills in the field of geriatrics medicine.

Our study was limited by the fact it was performed at a single academic institution. We involved several teachers, and cannot determine the effect of their own attitudes and style on the results, so generalizability of our findings is limited. Our study sample was relatively small, and we evaluated the effects of the intervention over a short period of time. Our study was not a randomized controlled trial; the two groups might not be fully comparable because we used alternation and alphabetical order of family name (quasi-randomization) to determine if individual students would be evaluated before or after the geriatric CST. However, we found no difference between the two groups for age and sex. It is also possible that other unmeasured factors, such as learning occurring independently of the CST, could have caused the observed attitude changes.

We believe our study has implications for future research. First, we suggest conducting studies with larger experimental group size, and evaluation over a greater time frame. Second, these studies should determine if including attitude-building elements in CST changes the attitudes of medical students towards geriatric patients better than our CST. Third, future studies could specifically address students with negative attitudes and determine the factors that improve their attitudes. Fourth, the modifiability of the different UCLA-GAS subscales by a teaching intervention could be evaluated in future studies.

## Conclusions

A geriatric skills-oriented educational intervention at the patients’ bedside may positively affect medical students’ attitudes towards elderly patients. However, in comparison to the impressive improvements in medical students’ knowledge and skills, the observed improvement of attitudes was small. Future skills-oriented educational interventions should take into account research findings that including attitude-building elements may enhance the effect of the geriatric CST on medical students’ attitudes.
